# Using geospatial techniques to develop an emergency referral transport system for suspected sepsis patients in Bangladesh

**DOI:** 10.1371/journal.pone.0191054

**Published:** 2018-01-16

**Authors:** Atique Iqbal Chowdhury, Rafiqul Haider, Abu Yousuf Md Abdullah, Aliki Christou, Nabeel Ashraf Ali, Ahmed Ehsnaur Rahman, Afrin Iqbal, Sanwarul Bari, D. M. Emdadul Hoque, Shams El Arifeen, Niranjan Kissoon, Charles P. Larson

**Affiliations:** 1 Maternal and Child Health Division, International Centre for Diarrhoeal Disease Research, Bangladesh (icddr,b), Dhaka, Bangladesh; 2 School of Public Health, University of Sydney, Sydney, New South Wales, Australia; 3 Department of Pediatrics, University of British Columbia, Vancouver, British Columbia, Canada; University of West London, UNITED KINGDOM

## Abstract

**Background:**

A geographic information system (GIS)-based transport network within an emergency referral system can be the key to reducing health system delays and increasing the chances of survival, especially during an emergency. We employed a GIS to design an emergency transport system for the rapid transfer of pregnant or early post-partum women, newborns, and children under 5 years of age with suspected sepsis under the Interrupting Pathways to Sepsis Initiative (IPSI) project.

**Methods:**

A GIS database was developed by mapping the villages, roads, and relevant physical features of the study area. A travel-time algorithm was developed to incorporate the time taken by different modes of local transport to reach the health complexes. These were used in a network analysis to identify the shortest routes to the hospitals from the villages, which were categorized into green, yellow, and red zones based on their proximity to the nearest hospitals to provide transport facilities. An emergency call-in centre established for the project managed the transport system, and its data was used to assess the uptake of this transport system amongst distant communities.

**Results:**

Fifteen pre-existing and two new routes were identified as the shortest routes to the health complexes. The call-in centre personnel used this route information to direct both patients and transport drivers to the nearest transport hubs or pick-up points. Adherence with referral advice was high in areas where the IPSI transport operated. Over the study period, the utilisation of the project’s transport doubled and referral compliance from distant zones similarly increased.

**Conclusions:**

The GIS system created for this study facilitated rapid referral of patients in emergency from distant zones, using locally available transport and resources. The methodology described in this study to develop and implement an emergency transport system can be applied in similar, rural, low-income country settings.

## Introduction

Emergency referral systems are imperative for countries where the population is heavily dispersed and resides in rural areas with poor transportation infrastructure [1,2]. A systematic and structured referral system ensures the rapid and timely transportation of patients to a health facility and increases the chances of receiving life-saving treatments [3]. An effective referral system must have a well-structured transport arrangement [4] to prevent health system-related delays [1,5–7]. These delays are easy to avoid with a swift, planned, and accessible transportation network within a referral system [4,8,9].

Socioeconomic factors influence how readily people with an income below the poverty line can access the health system [10], and geographical determinants are the strongest deterrent to the establishment of a well-connected referral transportation system [6,11–13]. UNICEF’s recommendations and toolkit for operating a perinatal referral transport service in rural India strongly advises the consideration of geographical condition (terrain and physical access) and the type of vehicles required to increase the efficiency of a referral transport system [14]. These recommendations also noted that in low- and middle-income countries, geographical aspects determine the accessibility of villages; what types of vehicles can navigate the roads to, from, and in those villages; and whether road improvement initiatives are required.

A geographic information system (GIS) is an effective tool for overcoming geographic barriers and identifying the fastest route to a health facility. The use of GIS have been quite prominent in a wide range of public-health applications [10,11,15–18], but few studies have applied GIS to develop or strengthen referral systems in low- and middle-income countries [7,19]. Studies that have employed GIS prioritised the identification of health service gaps and addressed the specific needs of their respective studied areas.

Sabde et al. [20] suggested that a GIS could improve the effectiveness of an emergency obstetric transport system in Madhya Pradesh, India. Their study detected hotspots of pregnant women, defined as locations at least 2 hours away from health services, to identify scopes of improving transportation facility. Sudhof et al. [12] identified potential gaps in accesses to emergency obstetric care in Rwanda by analysing the travel time of journeys made by ambulances. Bailey et al.[21] proposed a theoretical model of referral networks to reduce healthcare service gaps. Although, several similar studies have attempted to understand the accessibility, utilization, and cost-effectiveness of referral systems [2,3,10,22], there is a paucity of studies that have specifically focused on developing emergency transport systems using a GIS and, subsequently, evaluated its uptake amongst communities in distant and hard-to-reach areas. This study aims to fill this gap by demonstrating that a well-defined referral transport system can have a tangible impact on the willingness of distant communities to seek institutional healthcare.

First, we employed a GIS to develop an emergency referral transport system using the locally available infrastructure and resources. Second, we analysed the effect of introducing this transport system on the compliance with referral suggestions in availing hospital care amongst communities in distant and hard-to-reach areas. This paper is predominantly a description of the methodology used to develop a comprehensive GIS-based emergency referral transport system in a resource-constrained rural setting.

## Materials and methods

### Study setting and design

#### a) Study setting

**Fig 1:**
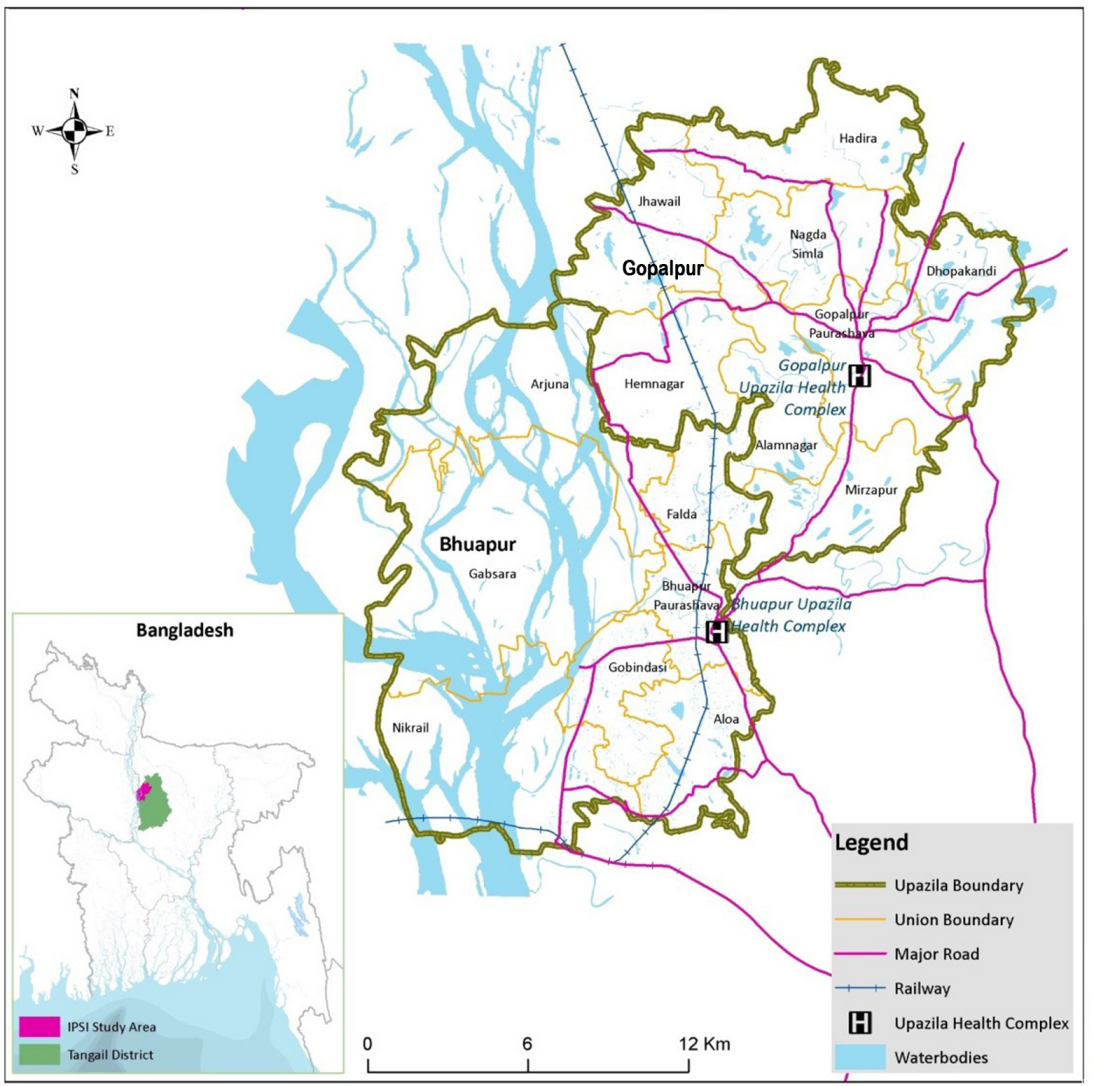
Project area: The Bhuapur and Gopalpur upazilas in the Tangail district. Inset map shows the location of the upazilas in Bangladesh.

The Interrupting Pathways to Sepsis Initiative (IPSI) project [23] was implemented in Gopalpur and Bhuapur, *upazilas* (analogous to subdistricts) in the Tangail district, located approximately 100 km northwest of Dhaka, the capital city of Bangladesh. The Gopalpur and Bhuapur upazila headquarters, approximately 45 km and 33 km away from the town of Tangail, respectively, is where the subdistrict hospitals—called *Upazila Health Complexes* (UHCs)—are located. Each subdistrict in the country has one such UHC. The map of the study area with locations of the UHCs is illustrated in Fig 1.

Gopalpur has a total area of approximately 194 km^2^ and a population of 252,331 [24]. The upazila administratively comprises 1 *pauroshova* (analogous to municipality), 7 *unions* (smaller units of subdistricts), and 194 villages [24,25]. Gopalpur is topographically flat, offering satisfactory access from the villages to the upazila headquarters. By contrast, Bhuapur has an area of around 227 km^2^ and a total population of 189,913 [24], comprising 1 pauroshova, 6 unions, and 144 villages [24,26]. Almost one-third of Bhuapur is hard-to-reach because of river islands, locally known as the *Chars*.

#### b) Study design: The emergency referral transport system

We designed a transport system for the rapid transfer of sepsis patients, as part of a larger referral system under the IPSI project. The IPSI emergency transportation system transported pregnant or early post-partum women, newborns, and children under five years of age with suspected sepsis to the Gopalpur or Bhuapur UHCs from a designated referral hub or sub-hub. Hubs were well-known locations within the two upazilas, where transport vehicles could be found, and were selected as pick-up points. Each hub was connected to reliable road networks and was coordinated by a designated transport manager, responsible for managing local transport at the transport hubs. These managers were trained to maintain the IPSI emergency transport.

The transport system was linked to and coordinated by a 24/7 ‘Sepsis Call-in Centre’ established for the project. The system’s purpose was to refer and guide suspected sepsis patients to the nearest transport hubs and sub-hubs for transfer to the nearest health facility. The call-in centre operator would assess the women and children based on a series of questions designed to determine the need for referral. If referral was required, the nearest hub was located, by searching the GIS database, and contacted by the transport managers to arrange transport for these patients. The system relied on local drivers and vehicles and included transport mediums, such as tempo, CNGs (three-wheeled auto-rickshaws run on compressed natural gas), and boats or trawlers. To ensure the vehicles were easy for the patients to recognize, each was equipped with an orange flag emblazoned with the project’s name on it. The IPSI emergency transportation vehicles brought patients directly to the hospital from the designated referral hub.

**Fig 2:**
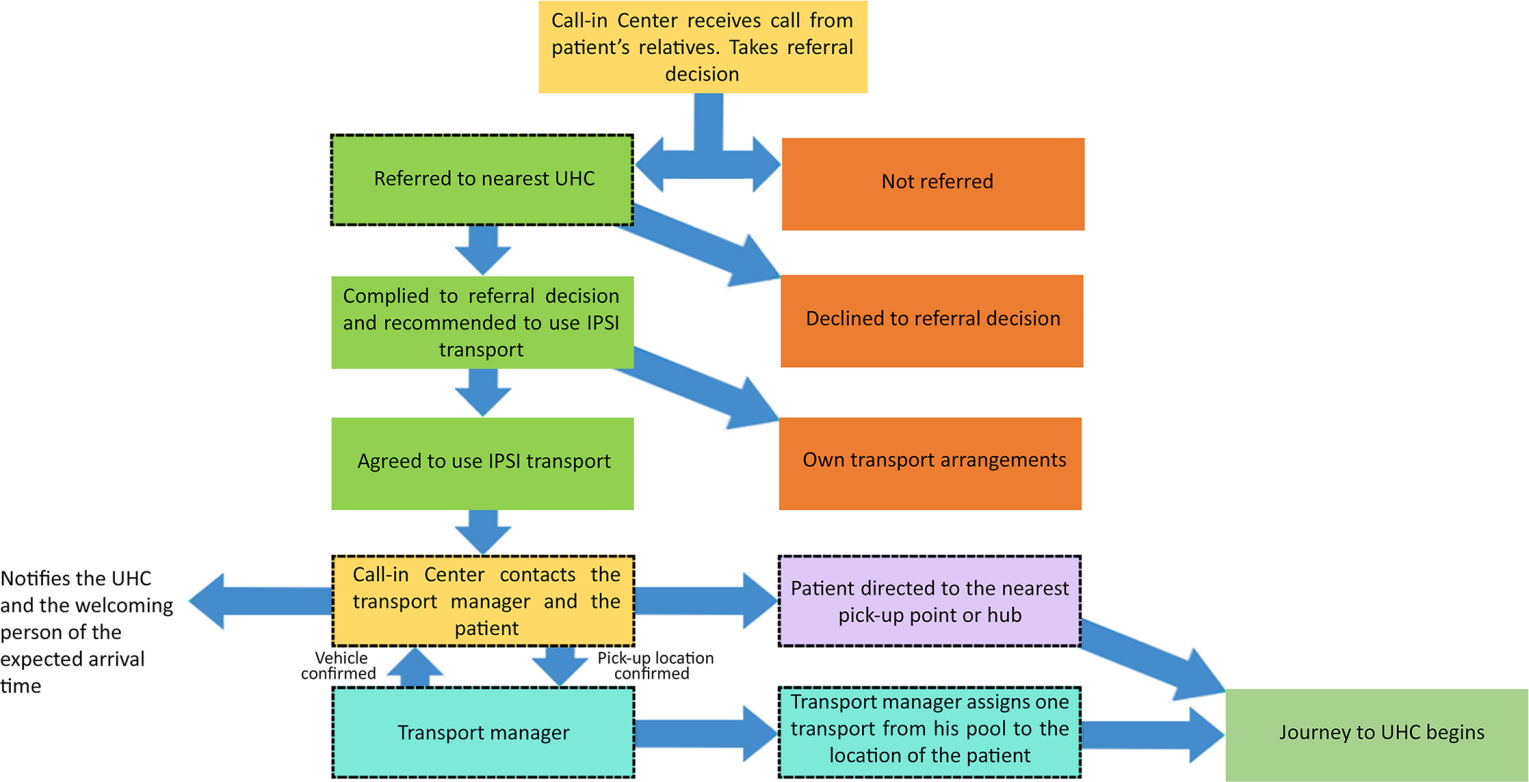
The operational schematic of the IPSI emergency referral transport system. Dotted lines represent the processes that directly employed information from the GIS database.

The patients’ arrival times were estimated for each journey. This time was communicated to the hospitals before a patient arrived; thus, preparations could be made to provide rapid treatment upon arrival. The system included transport assistance and reimbursements of the transport fare to the patients from distant zones, in accordance with the health facility’s catchment area, which was based on the estimated travel time. Further details about the nontransport components of the referral system can be found elsewhere [27]. Fig 2 provides an overview of how different processes were integrated to form the IPSI emergency referral transport system.

### Developing the emergency referral transport system

#### a) Creating the GIS database

The project required an accurate and detailed base map of the study area for the GIS modelling. A digital version of this map was obtained from the Local Government Engineering Department (LGED), Bangladesh, and was verified in the field via ground-truthing; a process of matching feature locations in maps with real world locations [28].

We found discrepancies between the collected map and the actual positions of union boundaries, road networks, settlements, and waterbodies. Moreover, certain areas were wrongly included in the study areas. The latitude and longitude values of many geographic features had significant differences between the map and real world because of spatiotemporal changes in the project area. Many of the roads, schools, mosques, rural markets, and other important landmarks were missing, as well. In addition, the map did not contain any village boundaries that were a prerequisite for the GIS analysis performed in this study. Therefore, the required correction and inclusion had to be performed through field data collection and verification.

**Fig 3:**
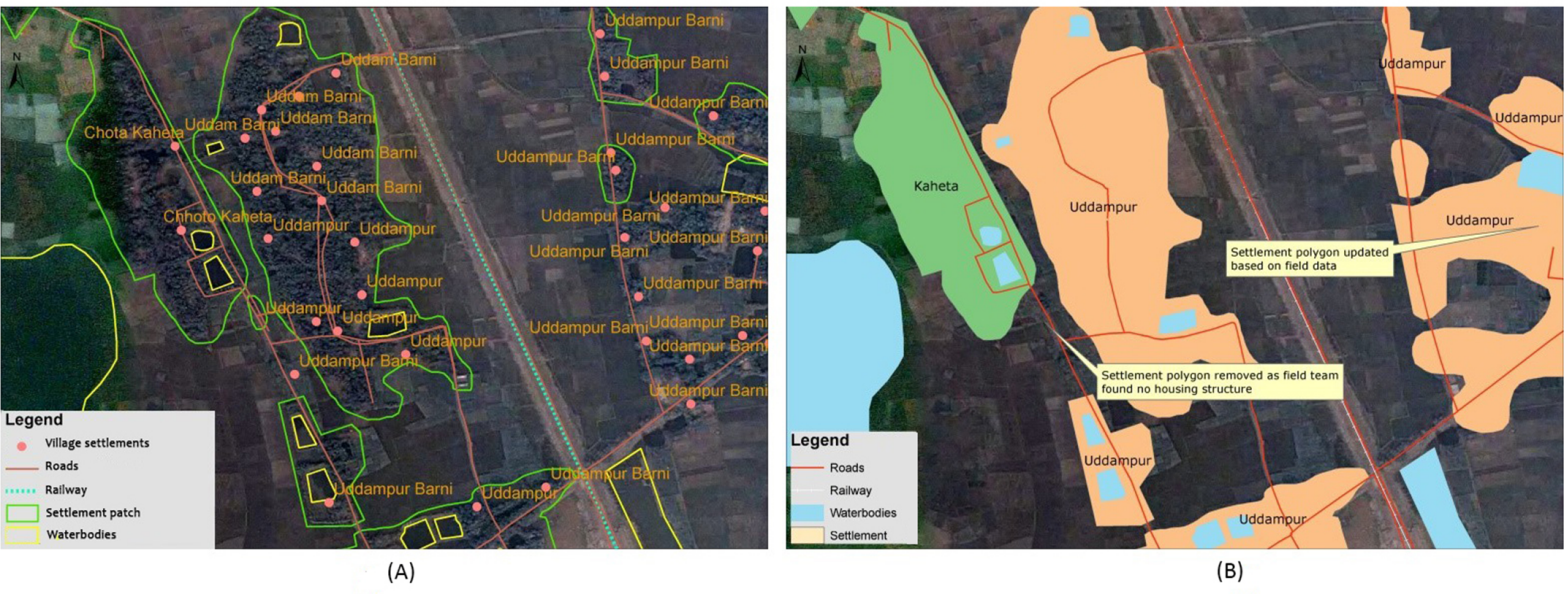
Base map preparation. (A) On-screen digitisation from the images to create the shapefiles. (B) Shapefiles created after on-screen digitisation.

A GIS database comprising high resolution satellite images and GPS receivers was created to correct the base-map anomalies. The project used 12 Garmin GPS receivers (eTrex Legend^®^ HCx and eTrex^®^ 30) to collect the latitude and longitude coordinates of the various features. The satellite images were extracted from Google Earth^®^ [29] and Landsat Thematic Mapper 5 from USGS EarthExplorer [30]. The images were then georeferenced, accordingly, by ground control point coordinates and digitised through an on-screen visual interpretation process to produce geographic features. In this process, all the geographic features were converted into a digital format and stored as layers with a unique identification number and name. After completing the digitisation process, the map features were printed and the mapping team then verified each map feature in the field. Fig 3 briefly illustrates the base map preparation.

i) Geographic features: Villages: The mapping team conducted in-person visits to every village from the census [24]. They recorded at least four coordinates at different corners of the settlement patches with a GPS and collected the settlement patches’ names. Next, the coordinates were overlaid on the digitised settlement features to ascertain the villages’ boundaries. The population data of each village were obtained from the 2011 census conducted by the Bangladesh Bureau of Statistics. Total population and household variables were then duly linked to the village boundary.

Waterbodies: The GIS database also contained rivers, canals, and ponds. These geographic features were extracted from Landsat TM 5, verified randomly on the ground, and incorporated into the maps when warranted.

Health facilities and pertinent landmarks: The GPS locations of health facilities, such as UHCs, private clinics at the upazila level, family welfare centres, and community clinics at the union level were collected. Similarly, the GPS coordinates and information on the pertinent landmarks, for example, education and religious centres, rural markets, transport (bus, tempo, rickshaw-van, CNG) stands, and landing stations were also collected and incorporated into the final map.

ii) Road network database and travel-time algorithm: Road network: The handheld GPS machines were used to prepare the road data while travelling across the study areas. To obtain consistent data, the cartography team travelled on the roads by riding in hired rickshaw-vans and walking (in places that were not accessible on a rickshaw) and travelled by boat on the water. The GPS’s auto tracking feature stored the moving positions with a date and time stamp, and the data were downloaded later for further processing. Next, the auto tracking GPS data logs were overlaid on the satellite images, and the final images were produced with the necessary adjustments for shapes and lengths. The team also collected the characteristics of the roads, such as type (paved-metal/herringbone and unpaved), name, and width, to be incorporated into the final images.

Transport and travel pattern: Roads and rivers were the two primary means of transport in the project area. On the mainland, most people travelled to the UHCs using a paddle rickshaw, rickshaw-van, CNG, or tempo. In the *Char* areas, people used boats to transport patients to the nearest g*hat* (landing stations), and from there, travelled by any one of the vehicles aforementioned.

Road vehicles were parked and availed from ‘transport hubs’, locally known as a tempo or CNG stand. Typically, one or two people (locally called the line/transport manager) monitored and managed the sequence of vehicles at these parking sites. These transport managers were contacted and then trained to communicate with the call-in centre prior to the implementation of the project transport system.

The tempo and CNG services from the upazila headquarters to different areas of the upazilas ran on fixed routes. Certain tempo and CNG services extended beyond the study’s upazilas. Mostly, these services connected the upazila headquarters (locally called *upazila sadar*) to prominent rural markets (locally called *hat* or *bazar*). Thus, each route had at least two transport hubs: one located at the community end and the other at the upazila sadar. Additionally, all the routes had several sub-hubs on the way to the upazila sadar as well as UHCs, where the vehicles would temporarily stop to drop-off or board passengers. These sub-hubs enabled patients to avail the transportation options near their villages, without having to travel to the nearest hub, which was still far away from their locations.

Rickshaws and rickshaw-vans were available throughout the project area, with no established stations. Wooden boats running on shallow engines (basically modified irrigation diesel pumps) were the only mode of transport between the Chars (small islands) and the mainland. However, these boat routes often changed, depending on the season. For example, during the wet season, the boat crossed the rivers in a relatively straight line; however, during the dry season, the boat crossed in curved and indirect paths that considerably increased the travel time. Walking was also a significant mode of transport, because the residents frequently walked short distances from their houses to the nearest transportation hub and adjacent areas of the UHCs.

Travel-time algorithm: A travel-time algorithm was developed to incorporate the mode of transport and road traversed, based on which the hubs nearest to the patient’s location was identified. Travel-time data (Table 1) were developed based on tempo, CNG, rickshaw-van, wooden boat, and walk time.

**Table 1:** Travel-time algorithm.

Mode of transport	Road type	Speed (km per hour)
Rickshaw/Rickshaw-van (three-wheeled pedal vehicle)	Paved (metal)	08.05
	Herringbone	08.55
	Unpaved	07.10
Tempo (three-wheeled autorickshaw run on diesel)	Paved (metal)	12.64
CNG (three-wheeled autorickshaw run on gas)	Paved (metal)	20.40
Walk	All type of road	04.00
Wooden boat (engine)	Waterway	08.00

We used a GPS to record the actual travel time and speed of the aforementioned transport modes on different types of roads and waterways. All the other roads (greater than or equal to 5 ft. wide), where the tempo and CNG services were unavailable, were computed based on rickshaw and rickshaw-van times. Walk time was employed for roads that were less than 5 ft. wide, because they were not accessible to vehicles. Next, this information was aggregated and assigned categorically for all the transport networks. The constraints on a transport network, such as a single-pole bamboo bridge (where only one person can walk across at a time) and natural barriers (river, canal, other wetlands) were also considered in the database.

This travel-time algorithm served two main purposes. First, it enabled calculating the travel time from the centre of a village to the hubs or sub-hubs. Thus, the place from where a vehicle should be dispatched to enable the earliest pickup could be identified. Second, the different travel times were used to estimate the patients’ arrival time at the health complexes. For example, if a patient used a rickshaw-van to travel the hub from home and then used the project’s CNG transport to reach the hospital, we used the distances traversed and the speed of vehicles in each segment of the journey to estimate the total time required to reach the UHC.

#### b) Network analysis

A network analysis was performed to develop the transport network system and define the catchment areas of the health facilities based on travel time. All the existing local transport services, both roads and waterways, were included.

i) Identification of the closest transport hub: The built-in *Closest facility* tool in the ArcMap module identified and calculated the route distances from the centre of a village to the nearest transport hub, and then to the closest health facility from the identified hub. This process helped the call-in centre operatives communicate with patients, the transport manager, and the staff receiving the patient at the hospital. When making the final choice about assigning a hub or sub-hub to a particular village in the system, the villagers’ opinions and input about proximity were considered. Consequently, a transport network was developed based on the shortest route distance, comprising well-defined routes from every village to the UHC. These routes were then assigned unique route identifiers to be used by the 24/7 call-in centre.

This transport network contained both pre-existing and newly introduced transport routes identified through the network analysis. The new routes were established to support a large portion of the areas where no existing transport services were available and added to the existing transport network (road and waterways) in the GIS database. The route that used a transport service with a small fleet of locally made three-wheelers called *Nasimans*, was excluded from the transport referral model. Riding in this specific type of transport is bumpy, which was very uncomfortable and risky for patient transportation. To compensate for any of the Nasiman routes, the villages covered by the excluded route were reassigned to other nearby routes. After defining the hubs and sub-hubs of the identified routes, each village was assigned to one hub or one sub-hub. In this process, proximity and physical accessibility from the hub to the centre of the villages were considered.

Some villagers were observed to use nearby UHCs in other upazilas, because their designated UHC was farther from their homes. During the catchment analysis, this situation was considered and transport routes were assigned accordingly. For instance, the routes were rearranged to enable the residents to attend the UHC closest to them, even if the UHC was in a neighbouring upazila. However, the residents of 18 villages in Bhuapur upazila travelled to nearby district hospital (other than the Tangail district hospital) that fell outside of the study area. Consequently, these villages were excluded from the study and resulting analysis.

ii) Defining the UHC catchment areas: The referral and transportation mechanisms were strengthened with a subsidy for the patients who were designated as “poor” and resided in distant regions. Therefore, the project area was divided into three zones in both upazilas, based on the respective UHCs that represented the catchment areas. The zones represented the physical accessibility of the project area and were measured based on rickshaws, because this mode of transport is the most common and widely available in the project area.

The *Service Area* tool of the network analysis was employed to define the catchment area of the facilities, based on travel time. We selected the UHCs as the facility points in the tool and generated multipart polygons based on 30- and 50-minute break values. The first zone, called the ‘green zone’, delimited the areas with less than or equal to 30 minutes of travel time and were considered easily accessible. The second zone, called the ‘yellow’ zone, was considered moderately accessible and required 30 to 50 minutes of travel time. The third zone, or the ‘red’ zone, was the most remote and difficult to access and comprised the villages beyond the 50-minute boundary. Finally, the villages were clipped using the multipart polygons to measure a villages’ catchment area (km^2^) and the proportion of the population that resided there. When a village was within the boundary of two polygons, we assigned it to the polygon, or zone, that covered 50% of the village.

**Fig 4:**
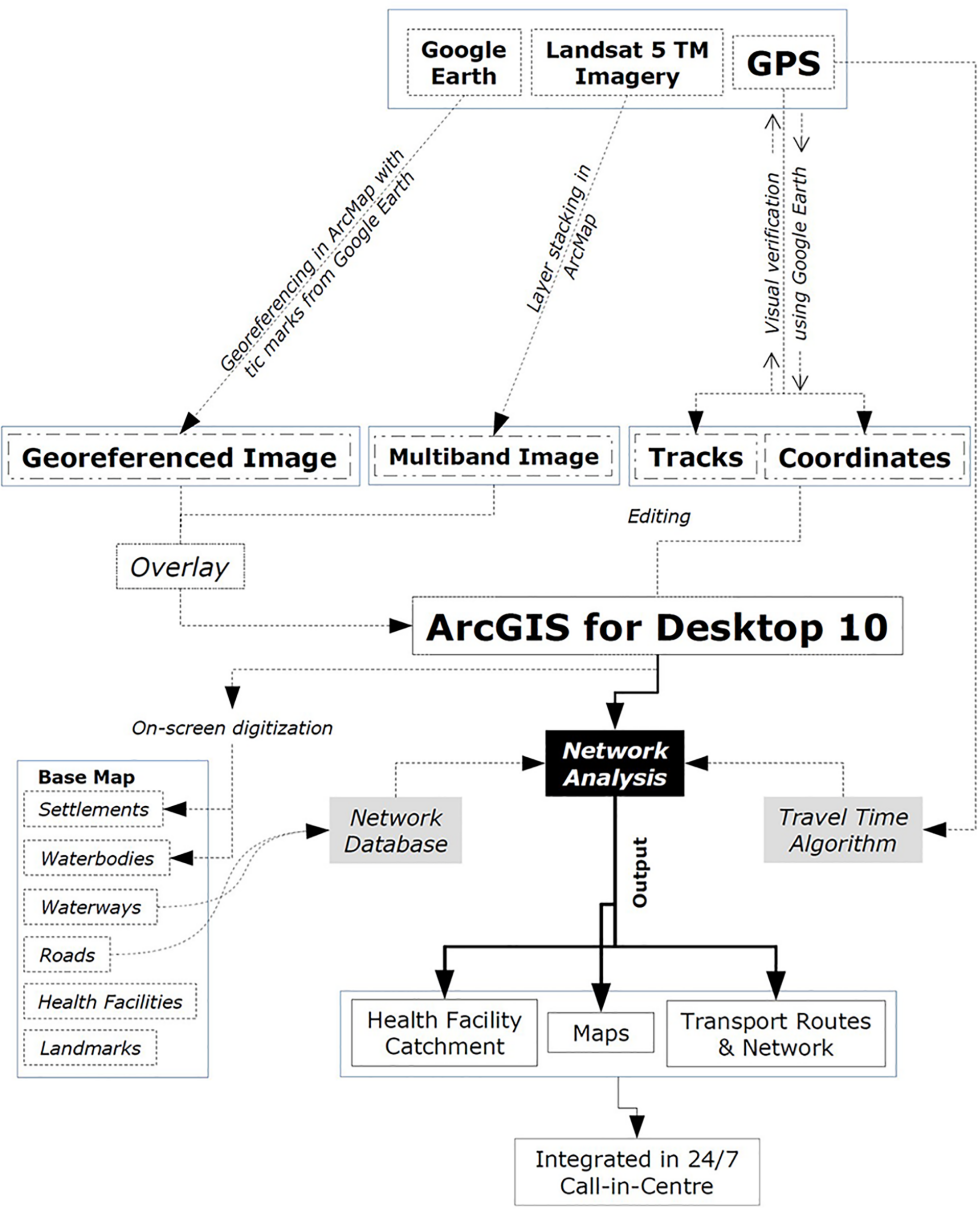
Flow chart explaining the full methodology of developing the transport model.

The transport model provided different means of financial support to cover transport fares and services, according to the zones. Typically, all the patients paid their own transportation costs; however, the patients designated as poor received monetary support. In the green zone, neither the transport facilities nor transport fares were provided from the project. In the yellow and red zones, a full-time (24/7) transport facility and needs-based reimbursements of transport fares were available for patients designated as poor. Fig 4 summarises all the steps in the development of the transport model.

## Ethics statement

This study assessed the uptake of a GIS-based emergency referral transport system in rural Bangladesh. The institutional Review Board of International Centre for Diarrhoeal Disease Research, Bangladesh (icddr,b), comprising the Ethics Review Committee and the Research Review Committee, approved this study (protocol number PR 14090). This study used only the absolute locations of the village centroids. The analysis did not involve interactions with human participants. Any individual information obtained was de-identified and used only as aggregated values. Therefore, the Review Board exempted us from obtaining consent.

## Data analysis

The IPSI call-in centre (operational from October 2013 to September 2015) data were evaluated to understand the effectiveness of the emergency referral transport system. The developed model attempted to reduce patient delays and provide a readily available transport mode for the residents in the distant parts of the study area. To assess the impact of this system on the willingness of people to avail institutional healthcare, we primarily focused on an analysis of the compliance with referral suggestions and use of the IPSI transport amongst residents in the yellow and red zones. The data were analysed by semester (6 months), to ensure equal interval comparability.

## Results

### a) GIS-based transport network

**Fig 5:**
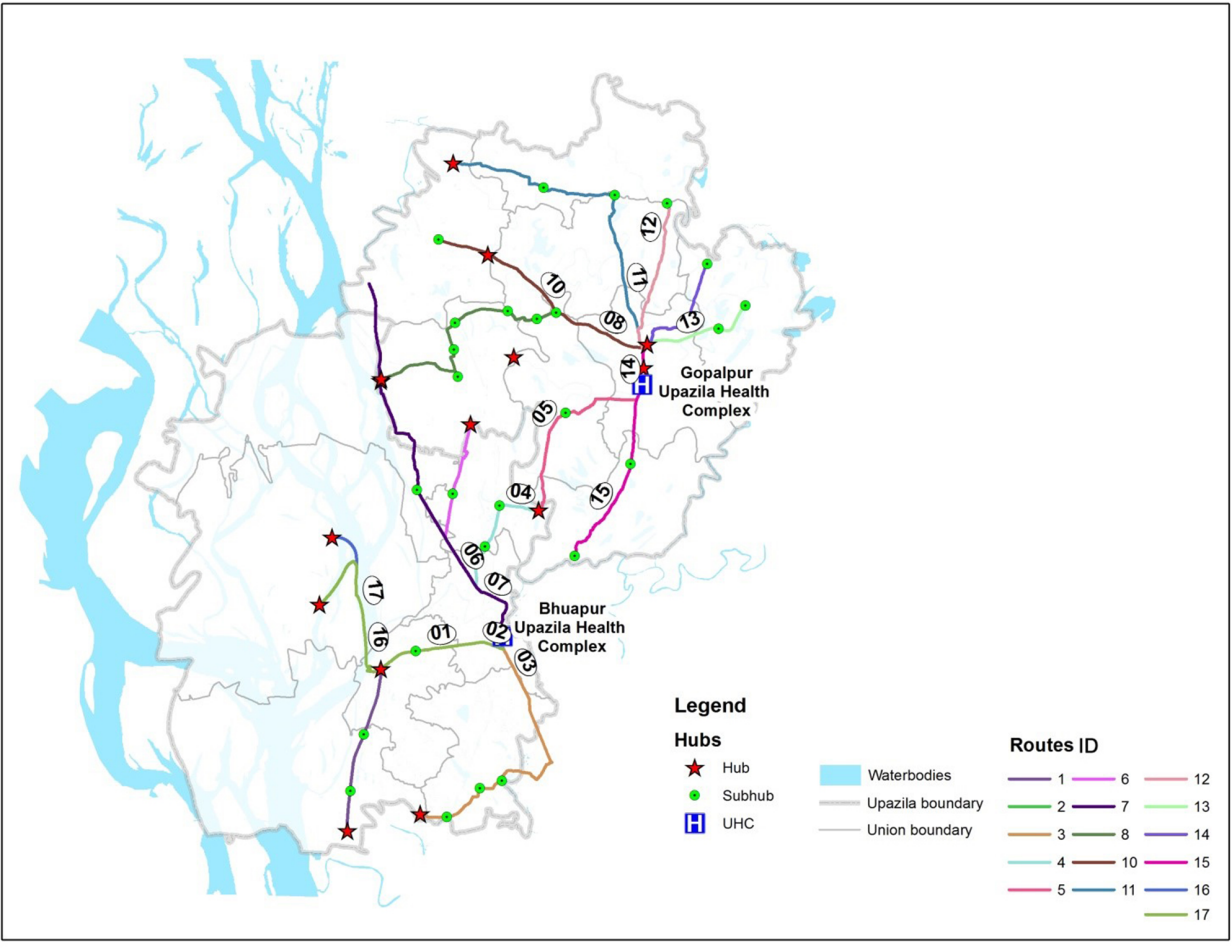
Transport network developed by the network analysis.

The transport system developed using the network analysis is illustrated in Fig 5, and the details of the individual transport routes are summarised in Table 2. Notably, some routes overlapped, because these routes followed the same roads to reach the UHCs. Fifteen pre-existing and two new routes were identified and included in the emergency referral transport network. These two new routes were Routes 12 and 15 (Fig 5), connecting residents from the unions of Nagda Simla and Mirzapur (Fig 1) to the Gopalpur UHC. Route 09 was not included in the final transport system because Nasiman was the main mode of transport along this route and posed a significant health risk to the patients. Upon further inspection of Fig 5, most routes had several sub-hubs that also acted as pick-up points.

**Table 2:** Description of routes.

Route ID	Route Name	Mode of Transport (Tempo/CNG/Boat)	Route Distance (km)	Route Travel Time (minutes)	Catchment Population
01	Shetu Bazar to Bhuapur UHC	Tempo	12.04	57.14	27,214
02	Gobindasi to Bhuapur UHC	Tempo	05.64	26.77	27,829
03	Nikrail Bazar to Bhuapur UHC via Shinguria	Tempo	11.92	56.59	14,497
04	Falda to Bhuapur UHC via Dighulia, Jhanjania	CNG	08.12	38.56	17,070
05	Falda to Gopalpur UHC via Alamnagar, Daulatpur, Suti Bazar	CNG	07.73	36.67	6,236
06	Sanak Boyra Bazar to Bhuapur UHC via Dobalia Bazar	CNG	10.10	47.94	16,791
07	Nalin Bazar to Bhuapur UHC	CNG	16.13	47.43	33,387
08	Nalin Bazar to Gopalpur UHC via Banglabazar, Hemnagar	Tempo	15.08	71.60	13,520
09*	Belua Bazar to Gopalpur UHC via Nabagram	Nasiman	08.34	39.61	7,547
10	Jhawail to Gopalpur UHC via Nabagram	Tempo	10.69	50.73	34,051
11	Bhengula to Gopalpur UHC via Chatutia Bazar Mod, Nagda Simla	Tempo	14.52	68.94	37,771
12	Konabari Bazar to Gopalpur UHC via Saydpur	Tempo	07.54	35.78	9,318
13	Konabari Bazar to Gopalpur UHC via Bhutia Bazar, Sajanpur Bazar, Madhupur upazila *sadar*	Tempo	06.26	29.70	17,983
14	Konabari Bazar to Gopalpur UHC via Pichuria, Dhopakandi, Dhanbari upazila *sadar*	Tempo	06.05	28.74	6,208
15	Konabari Bazar to Gopalpur UHC via Bara Shila, Suti Para, Mirzapur	Tempo	09.09	35.21	21,754
16	Ruli Para Trawler Ghat to Bhuapur UHC via Gobindasi Ghat	Boat and Tempo	17.35	80.38	7,120
17	Pungle Para to Bhuapur UHC via Gobindasi Ghat	Boat and Tempo	18.01	85.31	8,130

* Route ID 09 was later excluded from the transport network due to the risky nature of Nasiman transport.

**Fig 6:**
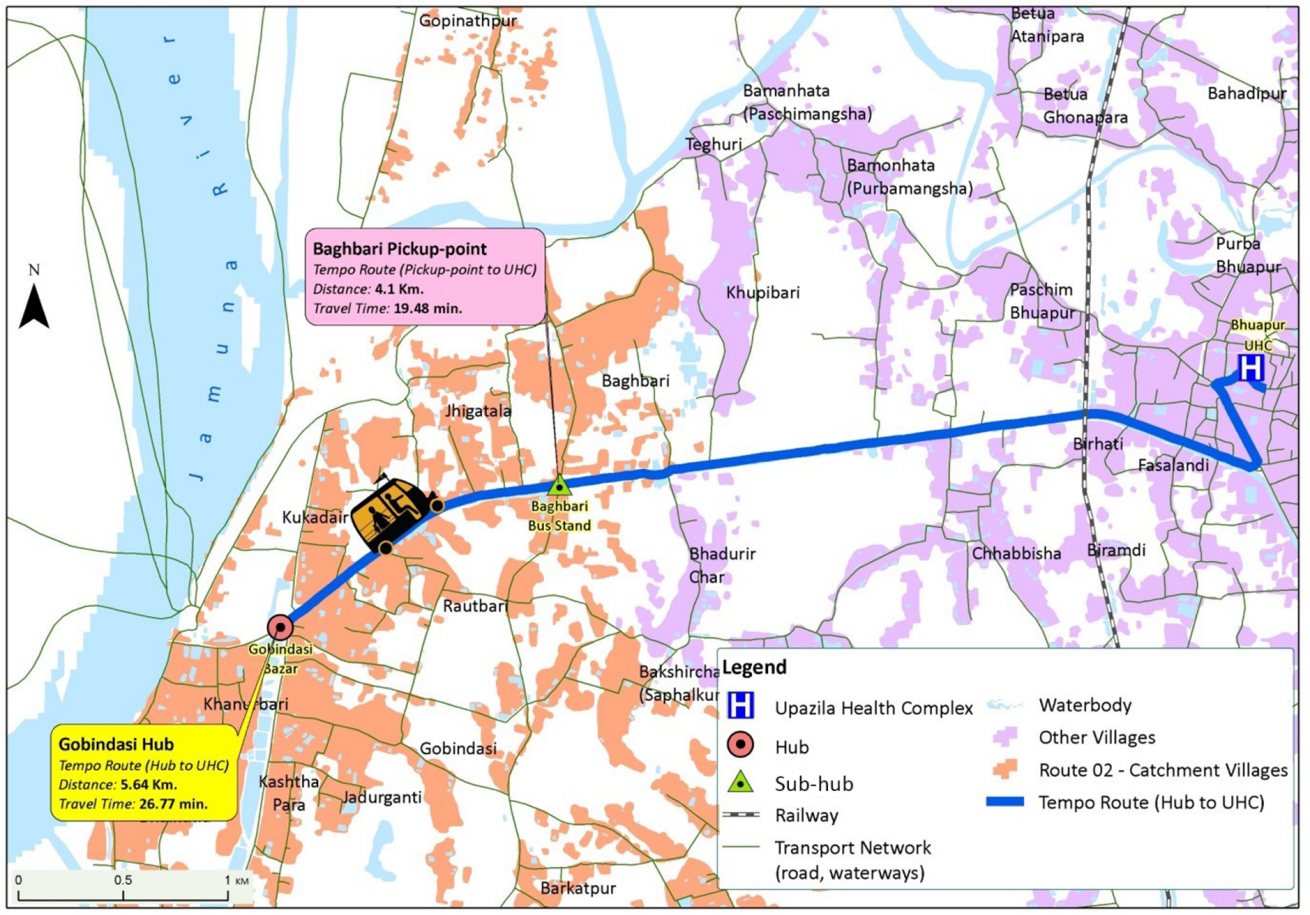
Defined catchment villages of the transport route.

The operational diagram in Fig 6 illustrates a single route and how the developed routes originated at rural markets or transport hubs and terminated at the desired UHC. Each route had a distinct catchment area (the villages shown in orange), several sub-hubs (e.g. the Baghabari pickup-point in Fig 6), and a transport hub. The sub-hub ensured that the patients from Jhigatala and Baghabari could avoid travelling back to the Gobindasi bazar or waiting with uncertainty for transport. The IPSI transport from the nearest hub (bazar end) could pick up patients on its way to the UHC.

**Fig 7:**
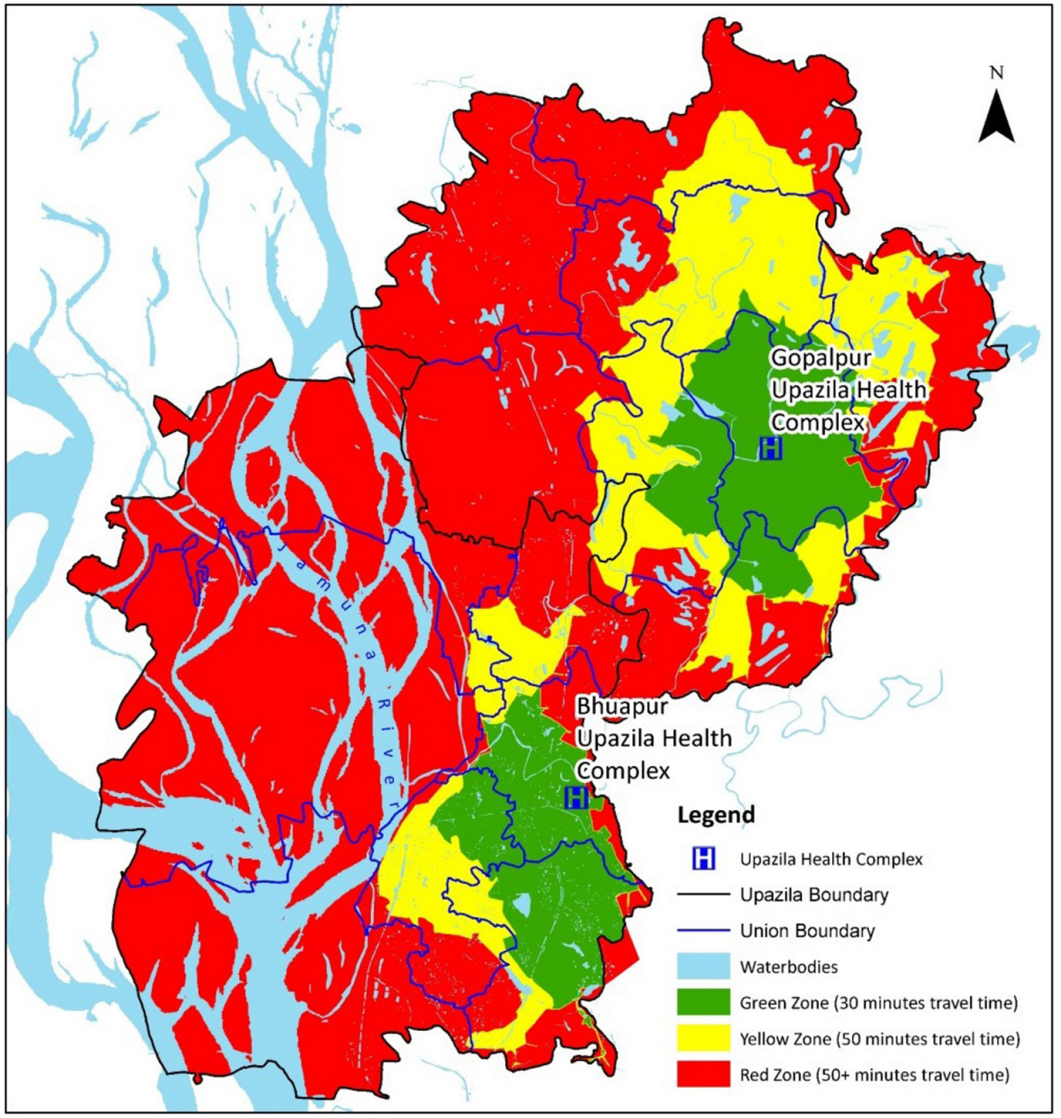
Delineating the zones based on travel time.

Table 2 also demonstrates that three routes (ID: 02, 13, and 14) had distance that required less than 30 minutes of travel time to reach the UHCs; seven routes (ID: 04, 05, 06, 07, 12, and 15) had a 30 to 50 minutes travel time; and the remaining seven routes (ID:01, 03, 08, 10, 11, 16, and 17), in the remote areas, were at a distance requiring greater than 50 minutes. These cut-off times divided the adjoining villages into three different zones, as represented by the three different colours on the maps in Fig 7.

The green zone comprised areas within 30 minutes of travel time, covering the entire pauroshova, some of its adjacent villages, and 24% of the total population (Table 3). The yellow zone, with its 30 to 50 minutes of travel time, was considered moderately accessible, and 28% of the population resided in this zone. Areas on the other side of the boundary that indicated a travel time greater than 50 minutes, were considered the most remote and covered 44% of the total population. The remaining 4% of the population residing in 18 villages in Bhuapur upazila, where the patients tended to travel to the nearby Sirajganj district hospital, were excluded from the study. Table 3 is a summary of the zones and their respective catchment populations.

**Table 3:** Travel time zoning with catchment area and population.

Travel Time	Zone	No. of Villages	Settlement Area (sq. km)	Population	Population (%)
30 minutes	Green	78	18.68	104,477	24
30–50 minutes	Yellow	111	25.23	123,110	28
50+ minutes	Red	131	38.02	195,112	44
Excluded from intervention and analysis		18	03.06	19,545	4
Total		338	84.99	442,550	100

### b) Evaluating the uptake of transport system: Call centre data analysis

During the study period (2^nd^ October 2013 to 29^th^ September 2015), 4,830 calls were made to the IPSI call-in centre from within the study area. Amongst these, nine referred calls were excluded from the database because the village and zone codes could not be ascertained. Of the remaining 4,821 calls, 2,731 calls (56.5%) were considered emergencies (suspected sepsis cases), and those patients were referred to the UHC for treatment. Table 4, summarises the data from the IPSI call-in centre database, which shows the status of the referred cases.

**Table 4:** Frequency of compliance with call-in centre referral advice and IPSI usage according to zone.

Semester	Reference Compliance				IPSI Used	
					No	Yes
First Semester (October 2013 – March 2014)	No	Zone	Green	30	30	-
			Red	162	162	-
			Yellow	135	135	-
		Total		327	327	-
	Yes	Zone	Green	91	91	-
			Red	330	225	105
			Excluded zone	1	-	-
			Yellow	322	306	16
		Total		744	621	123
Second Semester (April 2014 – September 2014)	No	Zone	Green	21	21	-
			Red	104	104	-
			Yellow	80	80	-
		Total		205	205	-
	Yes	Zone	Green	81	81	-
			Red	250	204	46
			Yellow	239	227	12
		Total		570	512	58
Third Semester (October 2014- March 2015)	No	Zone	Green	20	20	-
			Red	92	92	-
			Excluded zone	2	2	-
			Yellow	72	72	-
		Total		186	186	-
	Yes	Zone	Green	82	82	-
			Red	210	146	64
			Yellow	207	192	15
		Total		499	420	79
Fourth Semester (April 2015- September 2015)	No	Zone	Green	10	10	-
			Red	23	23	-
			Excluded zone	1	1	-
			Yellow	25	25	-
		Total		59	59	-
	Yes	Zone	Green	15	15	-
			Red	78	32	46
			Yellow	48	41	7
		Total		141	88	53

**Fig 8:**
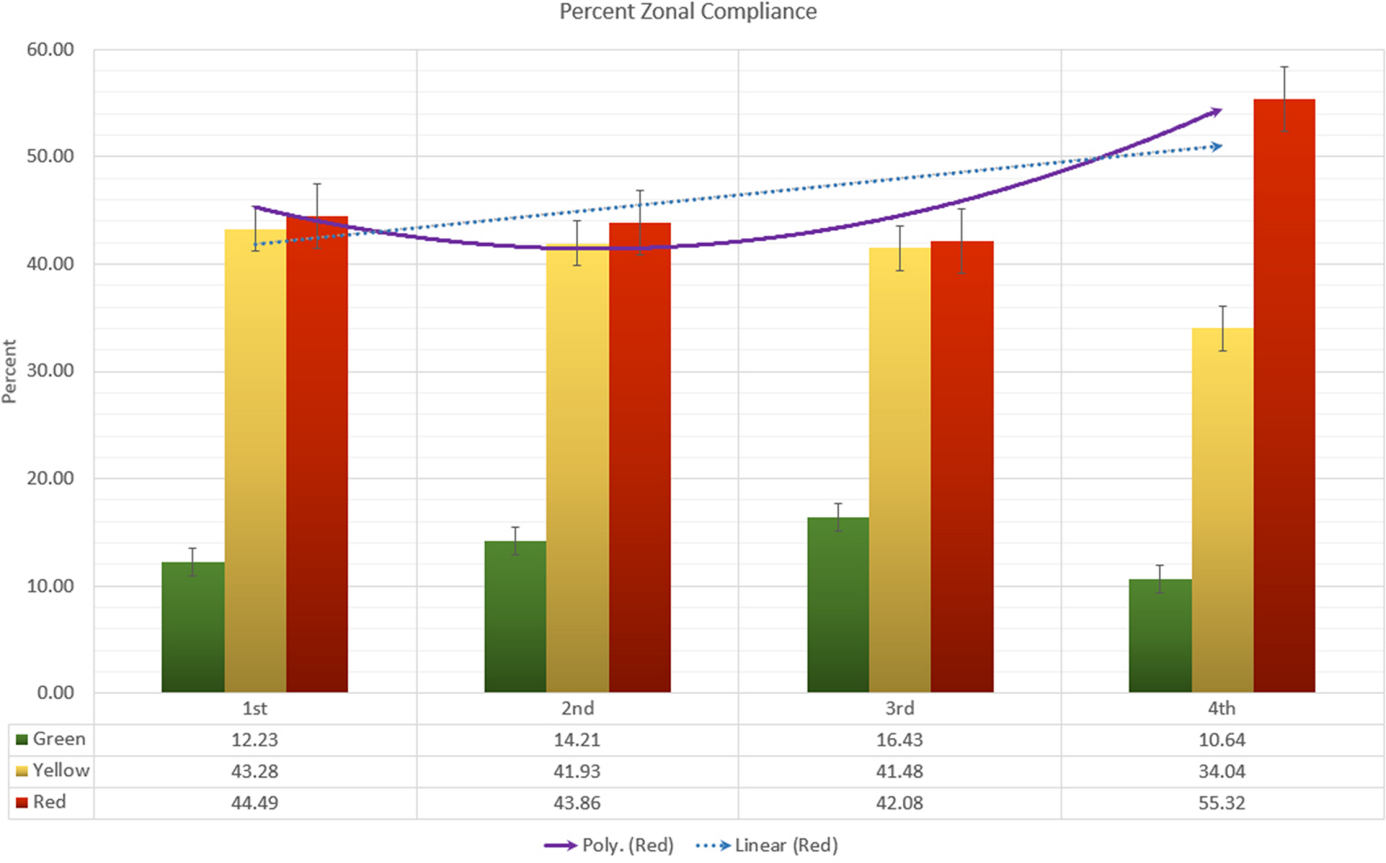
Percentage of zonal compliance with the referral advice during the four semesters. Polynomial and linear trend lines show the compliance pattern from the red zone.

Of the 2,731 referred patients, 1,954 (71.5%) complied with the referral advice, whereas 777 (28.5%) refused. Fig 8 illustrates the percentage of callers that complied with the referral decision during the project’s time period according to zone. Compliance with the referral decision remained low (less than 20%) in the green zone over the entire time period, whereas compliance in the yellow and red zones were much higher and, on average, three times that of the green zones. The red zones’ residents demonstrated the highest levels of compliance and use of the IPSI transport across every semester. Although, compliance from the green and yellow zones’ showed an overall net decrease from the first to last semester, the red zones showed an increase of 10.8%. The lowest percentage of compliance in the yellow (41.5%) and red (42.1%) zones were recorded in the third semester.

**Fig 9:**
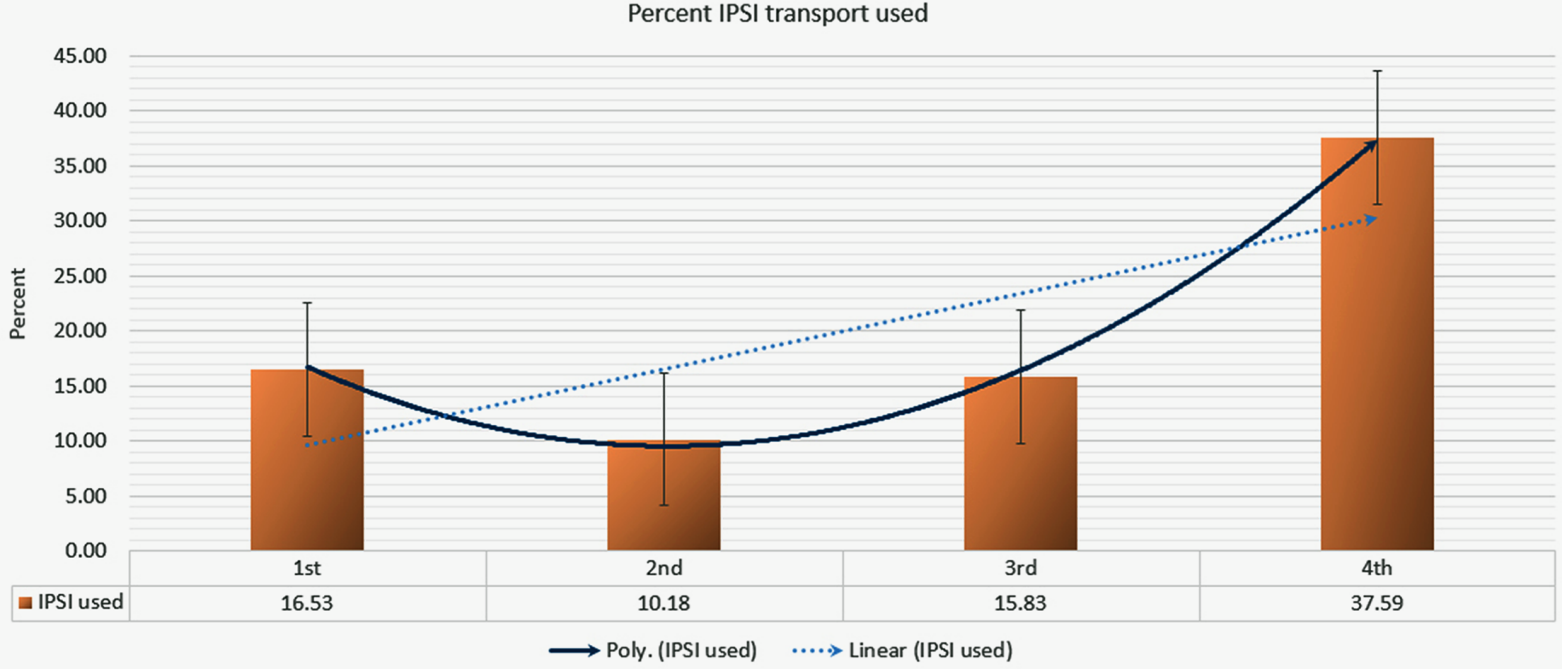
Overall percentage utilisation of the IPSI transport system over time.

Of the 1,954 patients that complied with call-in centre’s advice to go to a UHC, 313 patients (16%) used the IPSI transport system to reach that health facility. The remainder chose to arrange their own transport. Fig 9 illustrates the marked overall increase in the use of IPSI transport. Although the percentage of patients availing IPSI transport in the second semester showed a slight decline of 6.4%, utilisation in the last semester was 2.27, 3.70, and 2.30 times higher than the first, second, and third semester, respectively.

**Fig 10:**
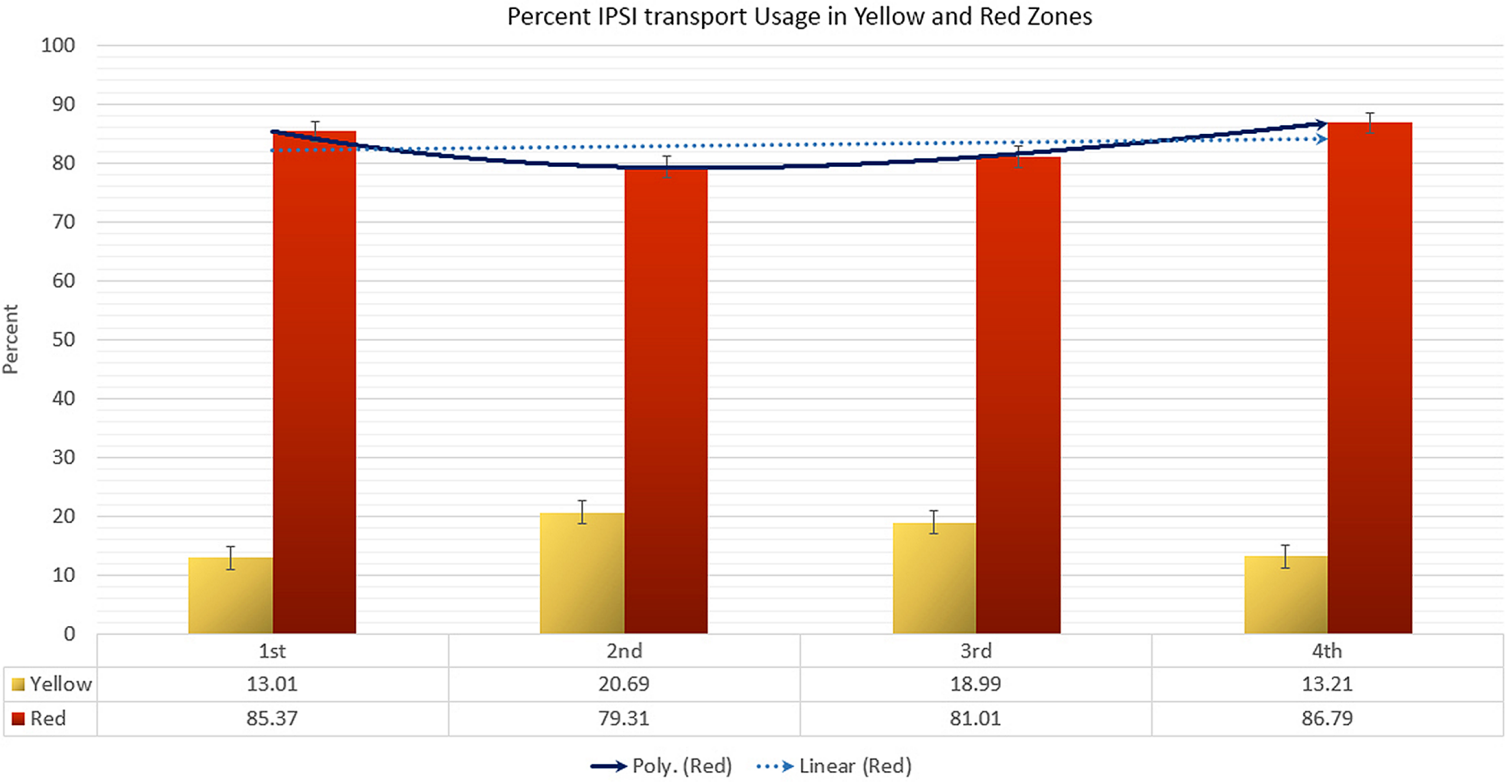
Zonal percentage utilisation of the IPSI transport system.

The zonal IPSI transport usage in Fig 10 presents an interesting pattern. Despite the same monetary support and service availability, the IPSI transport usage was considerably higher in the red zone than yellow zone. The percent of IPSI transport usage in the red zone was 6.56, 3.83, 4.27, and 6.57 times higher than the yellow zone during the first, second, third, and fourth semester, respectively. Although, the usage in the yellow zone alternated between increase and decrease, the usage in the red zone slightly decreased (0.07%) from the first to second semester but experienced a gradual increase in the final two semesters.

## Discussion

According to our review of the literature, we are the first to develop a GIS-based transport network for an emergency referral system in Bangladesh. We constructed a complete transportation system by generating spatial data and incorporating various transport modalities and travel time algorithms. We aimed to devise a transport system that considered the actual spatial organisations of certain features of the study area (settlements, roads, waterbodies, and important locations), and which could provide the fastest routes for transporting critical patients to the closest UHC.

Ensuring access to healthcare for people in low- and middle-income countries remains a challenge because most people live in areas with a dilapidated road infrastructure that contributes to the lower uptake of emergency healthcare services [31]. In addition to poor infrastructure, spatial attributes like rivers, canals, and other physical barriers result in lower access to healthcare services [1]. Despite this influence of spatial determinants on healthcare utilisation, the use of GIS remains heavily unexplored in the health sector of most developing countries [3,19,32].

Kim et al.[19] identified several causes for the underuse of GIS in Bangladesh, with the scarcity of spatial data and shortage of adequate knowledge amongst policy makers being the key reasons for its underutilisation. Although several past initiatives in Bangladesh, including *MaMoni* and BRAC *Manoshi* projects, focused on facilitating transportation within their referral systems, the use of GIS in developing these transport system was limited [33–35].

In the context of Bangladesh, our study provides crucial guidelines for devising a systematic emergency referral transport system within the prevailing transport system, which can utilise the existing transport and resources within a specific locality. Our study was implemented to specifically aid in the emergency transportation of patients with suspected sepsis. However, the approach and methodology we developed has wide-ranging application and is not limited only to transporting emergency patients. Our model could be extended to any initiative that requires a systematic transport system for serving any definite nonhealth related purpose.

In Bangladesh, the rural population is scattered throughout upazilas [36]. Thus, ensuring timely emergency ambulance services to such dispersed communities is extremely challenging. Currently, each UHC has a single ambulance which is not sufficient to cover its entire catchment area. Increasing the number of ambulances is not a feasible option because of the already existing challenges with the maintenance of these ambulances and unavailability of funds [37]. Additionally, these ambulances are not available or do not arrive on time during emergencies [38].

Integrating the existing transport system offers the flexibility of ensuring the continuous availability of transport services, and integrating a GIS with this initiative ensures identification of the fastest route. The purchase and maintenance costs associated with multiple ambulances could be avoided by using the local transport system, although the actual cost-benefit analysis warrants further research. In addition, many low-income countries have a challenging topography similar to Bangladesh and are subject to frequent seasonal changes [39]. While developing the IPSI transport network, we emphasised the geographic barriers (waterbodies), infrastructural setting (road type), and main mode of transport (rickshaw, van, auto-rickshaw, or walking) that existed in the study area. Furthermore, the emergency transport fleet comprised only vehicles that could withstand the adversities of the weather and climate and traverse the paved and unpaved rural roads.

Makanga et al.[40] showed that accessibility to hospitals was severely impaired due to unpaved roads becoming unsuitable for ambulance services. They demonstrated that the travel time was heavily influenced by seasonal changes; the road conditions declined during particular times of the year; and ambulances or similar vehicles could not easily reach the patient’s location. We minimised the effect of the seasons and topographical constraints on our system by considering a wide range of transport mediums when devising the IPSI emergency transport system. Developing an emergency referral transport system within an existing transport system has two major advantages. First, it focuses on utilising the existing facilities and logistical support in the area instead of adding expensive new additions into the system. Second, the use of local transport ensures a steady transport facility, reduces the overall cost of availing new resources, and ensures the selection of vehicles that can smoothly traverse the existing road types and waterways in the area.

An advantage of developing a GIS-based transport system is that it makes the identification of service gaps and routes unsuitable for transporting patients possible, compared with a non-GIS method [7,16]. Our GIS-based analysis identified one convenient, yet unsafe route (Route ID 09), because of our detailed assessment of the road type and mode of transport available. Catchment (health service coverage) analysis incorporating travel time facilitated a greater understanding of the distance that the villagers must travel to reach the most proximal UHC. Based on our data, we discovered that almost half the population resided outside the 50-minute travel-time zone, which also demonstrates the importance and necessity of an efficient transport system to assist these remote populations to rapidly reach health facilities during emergencies.

Most importantly, the IPSI emergency referral transport system demonstrated that with careful GIS-based planning, existing transport systems can increase people’s willingness to avail life-saving treatments. Call-in centre data revealed a very high referral compliance rate from the distant zones (yellow and red), where IPSI transport was readily available. A closer inspection also revealed an interesting temporal trend in the uptake of the IPSI transport, with the polynomial curve showing that the transport usage in the most distant red zone demonstrated a sharp increase. We observed a similar increase in compliance with the referral decision from the red zone during this period. Although we did not study the exact relationship between the use of IPSI transport and compliance, the availability of transport could have influenced compliance with availing healthcare. In the most-distant red zone, both its IPSI transport usage and compliance was higher than the yellow zone, suggesting that uptake and demand of such a transport system is greater amongst the more distant communities.

Machira et al.(2017) [41] showed that the availability of transport can motivate patients to seek healthcare in rural Africa and this result agrees with our observation. Ensor and Copper (2004) explored demand-side barriers to accessing health services that contributed to poor care-seeking practices during obstetric emergencies in Bangladesh [42]. Their findings suggested that reluctance to seeking emergency healthcare in Bangladesh was primarily because of poor communication systems, the very distant locations of health facilities, and the associated financial costs. In our study, the high compliance rate in the yellow and red zones coupled with the sharp increase in patients availing IPSI transport for reaching UHCs over time, supports the findings in earlier studies.

Further improvements to our described methodology are possible. First, availability of the exact village boundaries and centroid locations could increase the accuracy of the network analysis conducted in our study. If such administrative village boundaries are defined for Bangladesh in the future, our method would be more accurate in identifying the shortest routes. Second, our zoning approach considered only rickshaws while using the *Service Tool* in network analysis and the delineation would be further rectified if all the different transport modes could be utilised. However, for most routes, rickshaw was the main mode of transport, therefore representing the most common zonal picture of the study area. Third, a user feedback mechanism could be introduced to assess the flexibility and comfort while using the developed transport model. This assessment could detect the possible scope of improvements in the system required to increase system usage, which would not be generally detected by geospatial techniques. Fourth, we assessed the project’s call-in centre data to understand the transport uptake. Measuring the actual reduction in travel time by availing the project’s transport would have been more appropriate. This measurement was not possible because the project did not have a mechanism to measure the travel time of patients managing their own transport; thus, a comparison could not be made. Lastly, integrating the monsoon or seasonal effects into our transport analysis would have further strengthened our model. However, the data analysis was performed on a semester basis that balanced out the alternating dry and wet seasons during each semester. Thus, keeping the seasonal effect to minimum.

Despite the limitations, the major strength of this study is the use of GIS in developing an emergency referral transport system in a low-income country with a variable and complex geographical terrain. The methods described in this paper provide valuable guidelines for policy makers in Bangladesh to develop transport models that enhance the effectiveness of a referral system. Our proposed methodology has considered geographic variability and existing modes of transport and emphasised the use of inexpensive local resources. This method can be applied to create an organised, feasible, and locally accepted transport system for any region or to other development initiatives in Bangladesh.

## Conclusion

The use of GIS has been prominent in public health studies, but the application of this technology in the development of an emergency referral transport system has yet to garner attention in developing countries. This study, thus, offers valuable guidelines for devising a GIS-based transport system in a resource-constrained rural setting and can be adopted by any country with a similar context.

Developing an organised transport system can have critical implications in the context of a country like Bangladesh. The dispersed distribution of the population, shortage of resources, and financial constraints associated with establishing new facilities can be addressed by facilitating and enhancing the linkage of a community to existing health facilities. Remote communities have the greatest challenges to availing lifesaving healthcare services because of unreliable transport and geographical barriers. A structured transport system can increase care-seeking practices, reduce referral delays, and ultimately save lives. Operating a transport system using a dedicated call-in centre can ensure improved coordination and an increase in patient compliance. Integrating the emergency referral transport system with the existing transportation already availed by the local population can offer swift, stable, and readily available transport during emergencies.

## Supporting information

S1 File IPSI call in center data. (XLSX)

## Abbreviations

GIS: Geographic Information System
GPS: Global Positioning System
IPSI: Interrupting Pathways to Sepsis Initiative
UHC: Upazila Health Complex
